# Obituaries

**Published:** 1885-08

**Authors:** 


### OBITUARIES.

### The Late Dr. Leverett Bishop.

A special meeting of the Oneida County Homœopathic Medical Society was held at Dr. Wells' office to take action on the death of Dr. Leverett Bishop, of Sauquoit.

On motion of Dr. Watson, Dr. L. B. Wells was chosen chairman of the meeting. Dr. Wells, whose acquaintance with Dr. Bishop extended over a period of fifty years, offered the following memorial and resolutions:

"Dr. Leverett Bishop, one of the oldest persons in Oneida County, died in Sauquoit, Sunday, March 22d, aged ninety-three years and eight months. He retired the evening before in his usual health, but awoke at four A. M., and complained of pain in both sides of his chest. His wife gave him some remedies, but soon a decided change was manifest, and he said to his wife, 'I am dying.' He apparently did not breathe afterward, retaining perfect consciousness to the last. He was born in Guilford, Connecticut, but was brought when a child to Sauquoit, where the family afterward resided. His father died in 1866, aged eighty years. Dr. Bishop was educated at Hamilton Academy in Clinton, and studied medicine with Dr. Elnathan Judd at Paris Hill. His father served through the Revolutionary War, and when the second war commenced the son connected himself with the army. He went to Sacketts Harbor and served as surgeon's mate, an office which now corresponds to assistant surgeon. Dr. Bishop was intimately connected with the whites in negotiations with the Indians of that day, and was an intimate friend of the Indian Chief, Skenandoa. In 1816 Dr. Bishop came to Sauquoit to practice, and remained there until his death. The Doctor was by education and practice an allopath. His experience with drugs and observation of their uncertainties in relieving the sick led him to distrust the therapeutics of the dominant school. In 1844 and 1845 Dr. E. A. Munger at Waterville, Dr. Stewart at Clinton, and Dr. Haven at Hamilton became converts to Homœopathy, and Dr. Bishop was induced to give the system a thorough investigation. His acute and discerning mind soon grasped the great principle embodied in the law *Similia similibus curantur*, and in this his faith was unshaken to the end. His success in the new mode of treatment inspired him with a zeal in his profession which enabled him to overcome all obstacles. He continued to practice until ninety years old, when he was compelled to yield to the infirmities of old age. He was supposed to be the oldest practitioner in Central New York, if not in the State.

" His intellect was unimpared to the last. He was married to Miss Bacon, who bore him one child, a daughter, who is now the wife of Charles D. Rogers, superintendent of the well-known American Screw Company at Providence, R. I.

" In 1845, being left a widower, he married the widow of Dr. Rufus Priest. This lady survives him.

" In 1816 Dr. Bishop became a Mason, a member of the Chittenango Lodge. He was afterward a member of the lodge at Paris, and was one of the charter members of the Sauquoit Lodge of F. and A. M. in 1849. He was at his death an honorary member of the lodge. In 1833 he was ordained an elder of the Presbyterian Church in Sauquoit, and retained that office until his death, being the only survivor of six then dedicated to this office.

" His gentlemanly deportment and kindness to all will long be remembered by all who knew him, and especially by those who when suffering the pains and ailments of life were the recipients of his skill and experience.

" *Resolved*, That in the life of Dr. Bishop we recognize a kind Providence which has so long preserved a life of usefulness to his fellows.

" *Resolved*, That in the death of our colleague we lose a valued member of our Society, endeared to us by long associations, and as a Society and individually we shall cherish with grateful remembrance his fidelity to his professional principles and practice, and his virtues as a Christian gentleman.

" *Resolved*, That a copy of this memorial and resolutions be forwarded by the Secretary to the family of the deceased."

In moving the adoption of the memorial and resolutions, Dr. Watson spoke as follows:

"Mr. President and Gentlemen of the Society: It was my privilege to have known Dr. Bishop for more than thirty years. During that long period, covering nearly the lifetime of a generation of mankind, I have always found him the earnest and conscientious physician, the genial and courteous gentleman, the humane, benevolent, and honest man, ever faithfully striving to do what he considered most conducive to the welfare of his patients. None of the elder members of this Society, I am sure, ever forget the pleasant and beaming smile, the earnest and cordial greeting, and the warm and hearty clasp of the hand with which he met them. It is fitting that we should pause for a moment from the hurry and turmoil of the most exacting of the professions, and, standing beside his grave, should take a brief retrospect of the past, and borrow an example of well-doing from the life of the respected brother, who, after a useful and well-spent life, has passed onward, as we believe, to the better world."

The memorial and resolutions were adopted unanimously, and the Society adjourned.

---

New York, June 1st, 1885.

At a special memorial meeting of the Homœopathic Medical Society of the County of New York, held May 27th, 1885, the following resolutions were read and adopted:

"Whereas, It pleased Almighty God to lay aside from the active practice of his loved profession our esteemed associate, Benjamin F. Joslin, M. D., and lately to remove him from this life; therefore

"*Resolved*, That we bow to this providence, believing that he has found in the world beyond as certainly as in this that service for others constitutes one chief source of felicity.

"*Resolved*, That we recognize in the service of Dr. Joslin as an active member of this Society, as its presiding officer, as the Superintending Physician of the Five Points House of Industry, as a wise counselor in the emergencies of general practice, an earnest, enthusiastic, devoted physician, one who added lustre to the honored name he inherited, a Christian gentleman whose example we may emulate.

"*Resolved*, That a copy of these resolutions be sent to the family of our late colleague as an expression of our deep sympathy, and that copies be furnished our medical journals for publication."

A. B. Norton, M. D., Secretary.

NEW YORK, June 1st, 1885.

At a special memorial meeting of the Homœopathic Medical Society of the County of New York, held May 27th, 1885, the following resolutions were read and adopted:

"WHEREAS, In the recent death of John Butler, A. M., M. D., L. R. C. P., the New York County Homœopathic Medical Society has occasion to mourn the loss of an esteemed member; and,

"WHEREAS, It is befitting that this body should take suitable action to attest the feeling aroused among his professional associates by this untimely and most untoward event, therefore, be it

"*Resolved*, That, in our intercourse with Dr. Butler, we knew him as an earnest and laborious physician, whose bright and carefully trained intelligence had enabled him to attain to high rank in general medicine, and to pre-eminence in the special branch of electro-therapeutics.

"*Resolved*, That our acquaintance with him rapidly ripened into friendship, because by reason of his many attractive social qualifications and by reason of his high moral principles he impressed himself upon us as a man in whom affection and sincerity were conspicuous characteristics.

"*Resolved*, That in thus expressing its feeling, this Society desires to extend its sympathy to the family and friends of our lamented colleague.

"*Resolved*, That an authenticated copy of these resolutions be transmitted to Mrs. Butler, and that their publication be requested in our medical journals."

A. B. NORTON, Secretary.

